# Disparity between High Satisfaction and Severe Pain in Patients after Caesarean Section: A Prospective Observational-Controlled Investigation

**DOI:** 10.1155/2018/2634768

**Published:** 2018-04-11

**Authors:** Thomas Hesse, Andreas Julich, James Paul, Klaus Hahnenkamp, Taras I. Usichenko

**Affiliations:** ^1^Department of Anaesthesiology, University Medicine of Greifswald, Greifswald, Germany; ^2^Department of Anaesthesia, McMaster University, Hamilton, ON, Canada

## Abstract

**Objectives:**

Recent advances in the treatment of postoperative pain (POP) have increased the quality of life in surgical patients. The aim of this study was to examine the quality of POP management in patients after CS in comparison with patients after comparable surgical procedures.

**Methods:**

This was a prospective observational analysis in patients after CS in comparison with the patients of the same age, who underwent comparable abdominal gynaecological surgeries (GS group) at the university hospital. A standardised questionnaire including pain intensity on the Verbal Rating Scale (VRS-11), incidence of analgesia-related side effects, and incidence of pain interference with the items of quality of life and patients' satisfaction with the treatment of POP was used.

**Results:**

Sixty-four patients after CS reported more pain on movement than the patients after GS (*N*=63): mean 6.1 versus 3.6 (VRS-11; *P* < 0.001). The patients after CS reported less nausea (8 versus 41%) and vomiting (3 versus 21%; *P* < 0.001) and demonstrated better satisfaction with POP treatment than the patients after GS: 1.4 (0.7) versus 1.7 (0.7) (mean (SD); VRS-5; *P*=0.02).

**Conclusion:**

The disparity between the high level of pain and excellent satisfaction with POP treatment raises the ethical and biomedical considerations of restrictive pharmacological therapy of post-CS pain.

## 1. Introduction

New methods of analgesia and the organisational approach to analgesia, including the procedure-specific, multimodal analgesic protocols, may decrease the levels of postoperative pain (POP) and increase the quality of life of surgical patients [1–3]. Despite the advances in analgesia, caesarian section (CS) patients still experience high levels of postoperative pain in comparison with other surgeries [4, 5].

We recently measured the clinical effectiveness of the quality management system (QMS) based on procedure-specific, multimodal analgesic protocols, modified to meet the individual patient's requirements in our hospital [1]. The implementation of the QMS, based on the best existing evidence for treatment of POP in the form of PROSPECT [6] and DGAI [7] guidelines, led to the decreased levels of postoperative pain (POP), lower incidence of analgesic-induced side effects, and increased quality of life in treated patients [1].

However, only patients from the departments of general surgery, gynaecology, orthopaedics, and traumatology were included in the previous study [1]; the fact whether the patients from other departments enjoyed comparable clinical benefits in treatment of POP remains unknown.

Performing the annual audits, which are the part of our organisational measures to maintain a high quality of POP treatments and to improve it further, we have achieved a similar decrease in POP and analgesic-related side effects as well as increased quality of life in patients from all surgical departments, except for caesarean section (CS) patients, after QMS implementation (unpublished data).

To address this, we aimed to prospectively compare the results of audits in patients after CS with the data of women who underwent abdominal surgical procedures in the department of gynaecology at our hospital.

## 2. Methods

### 2.1. Study Design and Participants

The local institutional review board (ethics commission at the University Medicine of Greifswald) approved the protocol of this prospective observational investigation in January 2015 (approval no. BB 128/14). The investigation was performed using the data from the biannual audits within the frame of the QMS for POP [1] from the delivery room and gynaecology department at a university hospital of Greifswald.

The audits were performed during one month in spring each year, when 30 audit questionnaires per ward were distributed. All patients who were scheduled to elective CS were included. For the control group, patients aged 19–45 years who underwent gynaecological surgery (GS group) with an anticipated intensity of POP ≥3 from 10 on NRS-11 “a priori”-defined procedure-specific POP intensity depending on the size and location of surgical lesion [8] were included. Patients with chronic pain and patients with insufficient knowledge of the German language or with cognitive limitations were not included. The patients were informed that they will be enrolled in a routine quality assurance survey of the hospital and asked to fill in the questionnaire on the day of discharge.

### 2.2. Audit Questionnaire

The audit questionnaire was based on the questionnaire from our previous investigation [1] and included the following items: (1) if POP was present at all; (2) maximal pain intensity; (3) minimal pain intensity after surgery; (3) pain intensity on movement following surgery; (4) pain intensity on movement right now (on discharge from the ward); (5) whether pain disturbed the following aspects: sleep, mood, mobility, communication with others, and enjoyment of life; (6) whether the patient had received analgesics for more than 6 months before the surgery; (7) presence of tiredness, nausea, and vomiting; and (8) satisfaction with pain therapy.

All items concerning pain intensity were measured using the Verbal Rating Scale (VRS-11) from 0 = no pain to 10 = maximal pain imaginable. Satisfaction with the POP treatment was measured using the VRS-5 from 1 = excellent to 5 = bad, which, to facilitate the rating, resembles the scale of grades in German schools.

### 2.3. Perioperative Analgesia

**Figure 1:**
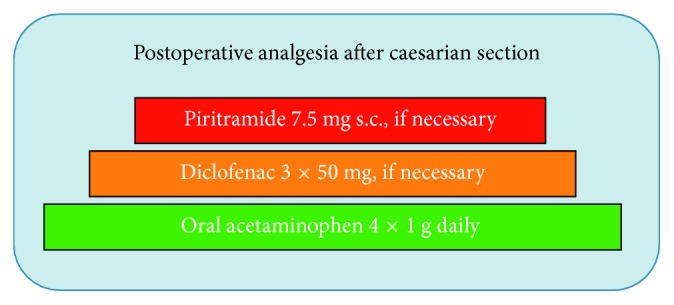
Scheme of multimodal analgesia adjusted to the expected level of postoperative pain in patients after caesarian section at the university hospital of Greifswald. Piritramide is an opioid analgesic with 0.7 potency of morphine, used to treat acute postoperative pain in Germany.

The standard anaesthetic technique for elective CS procedures in our institution is spinal anaesthesia with 7.5 mg hyperbaric bupivacaine and 5 mcg sufentanil, which provides sufficient analgesia maximally 4 hours after the surgery. The treatment of POP after CS includes oral acetaminophen 4 × 1 g daily supplemented by diclofenac 3 × 50 mg daily, if necessary. In case of insufficient analgesia, subcutaneous injections of 7.5 mg piritramide (opioid analgesic with 0.7 potency of morphine) were allowed up to 6 times daily (Figure 1).

The standard anaesthetic technique for GS was mainly general anaesthesia. For POP treatment following gynaecological surgery, basic analgesia was provided by the nonopioid analgesics acetaminophen, ibuprofen, and metamizole. In cases of expected moderate-to-severe pain, opioids were added, including oral tramadol or parenteral piritramide, applied via patient-controlled analgesia pumps. Continuous epidural analgesia with ropivacaine was used for POP treatment, where appropriate, according to abovementioned guidelines on POP treatment [6, 7].

### 2.4. Outcomes and Analysis

In order to compare the severity of pain after various surgical procedures, each type of surgery received the value of “a priori”-defined POP intensity, using the previously described procedure-specific POP intensity scale [8].

The intensity of pain taken on VRS-11, side effects of analgesia, quality-of-life items, and patients' satisfaction with their POP treatment were compared between the CS and GS groups. Using the IBM SPSS Statistics 24.0 software, these data were presented as mean and standard deviation (SD) of mean values. Continuous data were analysed using Student's *t*-test, and skewed data were compared using the Mann–Whitney test. Binomially distributed data were analysed using the chi-square test and presented as frequency distribution with absolute numbers and relative distribution in percent.

## 3. Results

The data of 64 consecutive patients after CS and 63 women of the same age who underwent other surgical procedures were available for analysis. The ASA physiological status was comparable among the patients of both groups (Table 1). During CS, spinal anaesthesia (SpA) was performed in 48 patients, epidural anaesthesia (EA) in 7, and general anaesthesia (GA) in 9. All patients from the gynaecological surgery (GS) group received GA for surgery, and 4 patients received EA in addition to GA. The detailed number of surgical procedures in patients from the GS group is given in Table 2.

**Figure 2:**
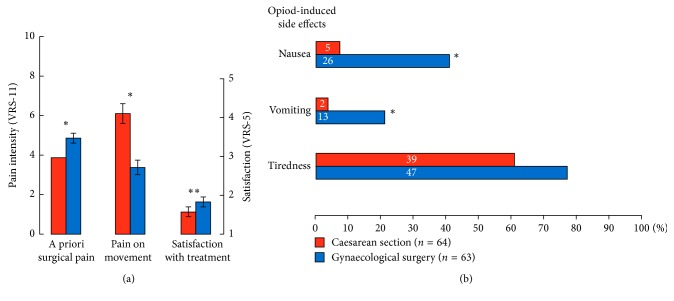
(a) Pain levels and patients' satisfaction with pain treatment. A priori surgical pain is an anticipated procedure-specific POP intensity, depending on the size and localisation of surgical lesion defined according to [2]. Data are given as mean (SD); ^∗^ *P* < 0.0001; Student's *t*-test. (b) Frequency of opioid-induced side effects given as percent from the total number of patients in the audits; numeric values within the bars on the graph are absolute number of patients; ^∗^ *P* < 0.0001; *χ* ^2^ test.

Although the anticipated intensity of POP (a priori-defined procedure-specific POP intensity, depending on the size and localisation of surgical lesion) was higher in the GS group, the patients after CS reported more pain on movement: 6.2 (2.0) versus 3.6 (1.9) (mean (SD)); *P* < 0.0001 (Figure 2(a); Supplementary Table (available here)). Other categories of pain measured during the audit (maximal, minimal, and pain) on discharge as well as the items of quality of life, disturbed by pain, were comparable among patients from both groups (Figure 2(b); Supplementary Table). The patients from the CS group reported less nausea and vomiting (*P* < 0.0001) than the patients scheduled to other surgical procedures (Figure 2(b); Supplementary Table). Patients after CS reported better satisfaction with the POP treatment than the patients after GS: 1.4 (0.7) versus 1.7 (0.7); *P*=0.02.

## 4. Discussion

Using the validated questionnaire for monitoring the quality of POP treatment at a university hospital, we have observed that, despite the higher levels of clinically relevant pain (pain on movement after the surgery), the patients after caesarean section (CS) were more satisfied with the treatment of POP than the patients after comparable gynaecological surgeries.

Our findings support the data from the previous cohort investigations, where high pain intensity scores were reported in patients after CS in comparison with patients after hysterectomy [4, 5]. The authors concluded that the unfavourable outcome was associated with the reluctance of opioid administration in patients after CS due to contradictory guidelines for treatment of POP in patients undergoing CS and for breastfeeding mothers [5].

The high levels of pain after CS in comparison with other surgical procedures were not astonishing since the pharmacological treatment of post-CS pain is restricted in the obstetrical wards worldwide (including our institution) due to peculiarities of the patients' category [9]. The main criteria for the choice of optimal postoperative analgesia for CS are the following: POP treatment should not interfere with the mother's ability to care for her baby and should be free from adverse neonatal effects in breastfeeding women. Thus, only few analgesic drugs match these requirements and are recommended for postoperative analgesia after CS [9, 10].

The gap between the severity of pain among the patients after CS and high levels of their satisfaction with POP treatment may be explained by both psychological and biological factors. Antenatal fears, especially concerning the health of the neonate, may worsen the severity of peripartal pain and then resolve after successful delivery [11]. On the other hand, the cognitive evaluation of pain severity during delivery is independent of the emotional feeling [12], which probably works as a part of the ancient reward system, including the endogenous antinociceptive circuits [13]. It is well known that delivery and CS alone elicit the profound response of pituitary proopiomelanocortin (POMC) system, resulting in elevated levels of beta-endorphins and ACTH, which may modify the emotional state of the patients after CS [14].

The extensive use of spinal anaesthesia for elective CS and restrictive use of opioids for treatment of post-CS pain in our institution are probably responsible for extremely low incidence of PONV in women after CS.

The limitations of our investigation are as follows: (i) small sample from the single-centre setting; (ii) questionnaire methodology (which has subjective nature in contrast to objective instrumental and laboratory measurements); and (iii) inhomogeneity of the control group (including both laparoscopic and laparotomic procedures), which can be improved in future studies. Moreover, such an outcome parameter as patients' satisfaction with postoperative pain treatment is subjective in its nature and is prone to bias in clinical trials, where participants are exposed to the factors, influencing their emotional sphere.

## 5. Conclusion

The disparity between the high levels of pain and excellent satisfaction with POP treatment with low incidence of analgesic-related side effects raises the ethical and medical questions of restrictive pharmacological therapy of post-CS pain.

However, regarding the recent findings about high risk of persistent pain development in patients with severe post-CS pain [15] and considering the ethical reasons (“pain is the fifth vital sign” [16]), the quality of POP treatment after CS should be improved by refining the multimodal analgesic approach and by introducing effective nonpharmacologic techniques in clinical routine [17].

## Figures and Tables

**Table 1:** Baseline characteristics.

	CS (*N*=64)	Other surgeries (*N*=63)	*P*
Age (years)	31 ± 5	33 ± 8	0.09
Body mass (kg)	89 ± 14	72 ± 16	<0.0001
ASA 1	21	20	1.0
ASA 2	38	42	0.51
ASA 3	5	1	0.26
Spinal anaesthesia	48	0	<0.0001
General anaesthesia	9	64	<0.0001
Epidural anaesthesia	7	4	0.3
Duration of surgical procedure (min)	37 ± 11	63 ± 31	<0.0001
Surgery-related “a priori”-assumed pain	4	4.8 ± 1.1	<0.0001

Data are presented as mean (SD) or as the number of patients; CS: caesarean section. Anticipated surgery-related “a priori”-assumed pain was taken using VRS-10, where 0 = no pain at all and 10 = worst pain which could be imagined according to [8].

**Table 2:** Other surgical procedures performed in female patients.

Surgical procedures	“a priori”-assumed pain (VRS-11)	Number of cases	Number of cases with malignancy
Abdominal hysterectomy	6	3	1
Abdominal hysterectomy with adhesiolysis	7	1	1
Colposuspension	5	1	0
Diagnostic laparoscopy	4	4	0
Laparoscopy-assisted vaginal hysterectomy	6	1	0
Laparoscopic supracervical hysterectomy	6	5	0
Laparoscopic adhesiolysis	4	4	0
Laparoscopic adnexectomy	4	5	0
Laparoscopic ovariectomy	4	22	1
Laparoscopic myomectomy	5	5	1
Laparotomy with adhesiolysis	8	3	0
Vaginal hysterectomy	5	9	0
Total	**4.8 (1.1)**	**63**	**4**

Data are presented as the number of patients and as mean (SD); expected surgery-related “a priori”-assumed pain was taken using VRS-11, where 0 = no pain at all and 10 = worst pain which could be imagined according to [8].

## Supplementary Materials

Supplementary Materials Supplementary Table: the intensity of pain, quality-of-life items, and patients' satisfaction with their treatment in patients after caesarean section and in patients after other surgical procedures.

## References

[1] Usichenko T. I., Röttenbacher I., Kohlmann T. (2013). Implementation of the quality management system improves postoperative pain treatment: a prospective pre-/post-interventional questionnaire study. *British Journal of Anaesthesia*.

[2] Tan M., Law L. S. C., Gan T. J. (2015). Optimizing pain management to facilitate enhanced recovery after surgery pathways. *Canadian Journal of Anesthesia*.

[3] Pogatzki-Zahn E., Kutschar P., Nestler N., Osterbrink J. (2015). A prospective multicentre study to improve postoperative pain: identification of potentialities and problems. *PLoS One*.

[4] Gerbershagen H. J., Aduckathil S., van Wijck A. J. M., Peelen L. M., Kalkman C. J., Meissner W. (2013). Pain intensity on the first day after surgery: a prospective cohort study comparing 179 surgical procedures. *Anesthesiology*.

[5] Marcus H., Gerbershagen H. J., Peelen L. M. (2015). Quality of pain treatment after caesarean section: results of a multicentre cohort study. *European Journal of Pain*.

[6] *PROSPECT Guidelines on Postoperative Pain Treatment*, October 2011, http://www.postoppain.org/

[7] *DGAI Guidelines on Postoperative Pain Treatment*, October 2011, http://www.awmf.org/uploads/tx_szleitlinien/041-001_S3_Behandlung_akuter_perioperativer_und_posttraumatischer_Schmerzen_aktualisierte_Fassung_04-2009_05-2011.pdf

[8] Jaffe R. A. (2010). *Anesthesiologist’s Manual of Surgical Procedures*.

[9] Lavoie A., Toledo P. (2013). Multimodal postcesarean delivery analgesia. *Clinics in Perinatology*.

[10] Siddik S. M., Aouad M. T., Jalbout M. I., Rizk L. B., Kamar G. H., Baraka A. S. (2001). Diclofenac and/or propacetamol for postoperative pain management after cesarean delivery in patients receiving patient controlled analgesia morphine. *Regional Anesthesia and Pain Medicine*.

[11] Fenwick J., Hauck Y., Downie J., Butt J. (2005). The childbirth expectations of a self-selected cohort of Western Australian women. *Midwifery*.

[12] Salmon P., Miller R., Drew N. C. (1990). Women’s anticipation and experience of childbirth: the independence of fulfillment, unpleasantness and pain. *British Journal of Medical Psychology*.

[13] Leknes S., Tracey I. (2008). A common neurobiology for pain and pleasure. *Nature Reviews Neuroscience*.

[14] Harbach H., Antrecht K., Boedeker R. H. (2008). Response to delivery stress is not mediated by beta-endorphin. *European Journal of Obstetrics & Gynecology and Reproductive Biology*.

[15] Weibel S., Neubert K., Jelting Y. (2016). Incidence and severity of chronic pain after caesarean section: a systematic review with meta-analysis. *European Journal of Anaesthesiology*.

[16] Lanser P., Gesell S. (2001). Pain management: the fifth vital sign. *Health Benchmarks*.

[17] Hesse T., Henkel B., Zygmunt M., Mustea A., Usichenko T. I. (2016). Acupuncture for pain control after caesarean section: a prospective observational pilot study. *Acupuncture in Medicine*.

