# Supplementary material for: Disparity between High Satisfaction and Severe Pain in Patients after Caesarean Section: A Prospective Observational-Controlled Investigation
Source: Anesthesiol Res Pract. 2018 Apr 11;2018:2634768. doi: 10.1155/2018/2634768 (PMC5925022; doi:10.1155/2018/2634768)
Supplement: Supplementary Materials — Supplementary Table: the intensity of pain, quality-of-life items, and patients' satisfaction with their treatment in patients after caesarean section and in patients after other surgical procedures. [file 2634768.f1.pdf]

Supplementary table.

	CS (N=64)	Other surgeries (N=63)	***P***
Pain intensity (VRS-11)			
maximal pain	6.1 (2.1)	6.0 (2.7)	0.82
minimal pain	1.9 (1.7)	2.1 (1.6)	0.49
on movement	6.2 (2.0)	3.6 (1.9)	<0.0001
on discharge from the ward	2.1 (1.6)	1.8 (1.1)	0.22
Satisfaction with therapy (VRS-5)	1.4 (0.7)	1.7 (0.7)	0.017
Pain disturbed:			
general activity*	92 (62)	50 (84)	0.16
mood*	45 (29)	38 (22)	0.5
sleep*	65 (42)	67 (42)	0.71
enjoyment of life*	43 (27)	43 (23)	0.97

Data presented as mean (SD) or as * per cent (absolute number of patients); CS – caesarean section; VRS - Verbal Rating Scale (explanation in text)
